# Supplementary material for: Chromothripsis during telomere crisis is independent of NHEJ, and consistent with a replicative origin
Source: Genome Res. 2019 May;29(5):737–49. doi: 10.1101/gr.240705.118 (PMC6499312; doi:10.1101/gr.240705.118)
Supplement: Supplemental Material [file supp_gr.240705.118_Supplemental_file_1.zip › contigs/annotated_contigs/DB115/contig.2.DB115_length_289_mean_cov_3.63321799308.docx]

**DB115_length_289_mean_cov_3.63321799308**

```
GTCAGGATTTTAAACATGCTTGAGAGTTTCTCATGAATACCATCAGAAGAGC|AGCT|TAATTTAATTATGGAAAAGGTCTTTATAATT
 >chr9:19659826-19659882 + E=2e-22                   >chr9:19592241-19592478 + E=9e-132
TTTTTGCCCTTGATATCACAACTTTTCTACCATTTTCTCCCTTATACAAATGAACAAGGATTGACTTTTTAAATAGTGGTGAAATTATT

TTTCTGGAAATGTCTTTGGTTAAATGGCAGAGAGTTTAGGACAATACAATGGAGACAGCTTTCCCCACATATTGCTTCATTGCAAGATA

TTAGTTGGGATATCTACCGACCTA
```
